# Prevalence and molecular characterization of alpha and beta-Thalassemia mutations among Hakka people in southern China

**DOI:** 10.1590/1678-4685-GMB-2022-0043

**Published:** 2022-10-24

**Authors:** XiangXing Zeng, ZhiFang Liu, CaiHua He, Jia Wang, LiXiang Yan

**Affiliations:** 1 Heyuan Women and Children’s Hospital, Laboratory of Medical Genetics, Heyuan, Guangdong, China.; 2 Heyuan Women and Children’s Hospital, Department of Clinical Laboratory, Heyuan, Guangdong, China.; 3 Heyuan Health Supervision Institute, Department of Integrated Enforcement, Heyuan, Guangdong, China.

**Keywords:** Genetic mutations, Thalassemia, Hakka, Southern China

## Abstract

Our aim was to investigate molecular features of thalassemia for proper clinical consultation and prevention in Heyuan. In our research, a total of 25,437 positive screening subjects were further subjected to a genetic analysis of α-thalassemia (α-thal) and β-thalassemia (β-thal). The deletion of α-thal mutation was tested by Gap-PCR, while the non-deletion of α-thal and β-thal mutation were identified by the PCR-reverse dot blot (PCR-RDB) technique. Nested PCR detected Hkαα/-- ^SEA^ and Hkαα/αα. Among the 25,437 positive screening subjects, 44.09% (11216/25437) subjects were bearers of thalassemia variations, and 30.85% (7847/25437) subjects showed α-thal changes alone. Among the 23 genotypes with α-thal mutation alone, the three common genotypes were --^SEA^/αα(68.34%), -α^3.7^/αα(16.44%), and -α^4.2^/αα(6.38%). Of the 11.50% (2924/25437) subjects and 29 genotypes with β-thal mutation alone, the three common genotypes were β^CD41-42^/β^N^(36.22%), β^IVS-II-654^/β^N^(30.88%), and β^-28^/β^N^(13.47%). Additionally, of the 1.75% (445/25437) subjects and 55 genotypes showed both α- and β-thal mutations. We also identified 269 cases of Hb H and six patients of Hkαα. Furthermore, the common genotypes of α-thal and β-thal mutations were consistent with allele frequencies of mutations. Our study establishes molecular features of thalassemia among Hakka people in Heyuan. It will be useful for developing strategies to prevent thalassemia.

## Introduction

Thalassemia is an inherited chronic hemolytic disease on account of the deletion or point mutation of the human globin gene. According to the type of defective gene, thalassemias are divided into α-, β-, γ-, δ-, δβ-, and εγδβ-thalassemias. Among them, α-thalassemia (α-thal) and β-thalassemia (β-thal) were the two main types (Barnett, 2019; Shafique *et al*., 2021; De Simone *et al*., 2022). Carriers of thalassemia mutations account for about 5% of the global population, particularly in tropical and subtropical areas, for instance, North Africa, the Mediterranean region, India, Southeast Asia, Pakistan, and the Middle East (Merkeley and Bolster, 2020). In China, thalassemia is mainly found in the south of the Yangtze River, especially in the Guangxi, Hainan, and Guangdong regions (Yang *et al*., 2019; Cheng *et al*., 2022).

Human hemoglobin has three different developmental stages in the embryo, fetus, and adult. At the molecular level, hemoglobin synthesis is determined by two multigene clusters which are located in chromosome 16 and chromosome 11, respectively (Merkeley and Bolster, 2020; Shafique *et al*., 2021). The underlying molecular defects in the α-globin or β-globin gene clusters constitute the foundation of hemoglobin synthesis defects and the various genetic forms of α- or βthal (Taher *et al*., 2018; Viprakasit and Ekwattanakit, 2018). According to the severity of clinical symptoms, thalassemia is primarily divided into thalassemia trait (TT), thalassemia intermedia (TI), and thalassemia major (TM). TI and TM are generally identified as thalassemia patients (Taher *et al*., 2018; Viprakasit and Ekwattanakit, 2018). The heterozygous state of α⁺-thal (αα^T^/αα,-α/αα,α^T^α/αα) or α^0^-thal (-α/-α, --/αα,-α/αα^T^) is general clinically microcytic anemia or asymptomatic. While α-TI also called hemoglobin H disease (Hb H) (--/-α, --/α^T^α) is usually due to the compound heterozygous types for α⁺-thal and α^0^-thal. The homozygous types for α^0^-thal lead to hemoglobin Bart’s (--/--), which gives rise to death *in utero* or shortly after birth (Farashi and Harteveld, 2018; Kalle Kwaifa *et al*., 2020). The β-thal heterozygous state (β^+^/β^N^, β^0^/β^N^), which is called β-thalassemia trait or β-thalassemia minor, usually presents as asymptomatic microcytic anemia. Both β-thalassemia intermedia (β-TI) and β-thalassemia major (β-TM) arise from compound heterozygotes or homozygous β-globin mutations. Patients with β-TM usually depend on blood transfusion throughout life because they suffer from severe anemia since infancy. In contrast, patients with β-TI show considerable heterogeneity and may have mild or moderate anemia, and thus have different blood transfusion requirements (Jaing *et al*., 2021, Taher *et al*., 2021).

The clinical phenotype of thalassemia ranges from asymptomatic to fatal hemolytic anemia. So far, studies have shown that hematopoietic stem cell transplantation (HSCT) can cure thalassemia. Due to family economic conditions, post-transplant responses, and donor sources, a large number of patients still choose to receive long-term, massive blood transfusion therapy to inhibit the production of erythropoietin, reduce the incidence of skeletal deformities, and maintain the normal growth and development of patients with TM (De Simone *et al*., 2022). For that matter, it is significant to implement the genetic testing of thalassemia in the population of childbearing age and prenatal diagnosis for high-risk groups of thalassemia in the first and second trimesters to prevent the birth of the fetus with TM. As of May 2022, more than 948 mutant forms of the β-gene and 827 mutant forms of the α-gene have been identified, which are described in detail in the HbVar database (https://globin.bx.psu.edu/)

**Figure 1 -:**
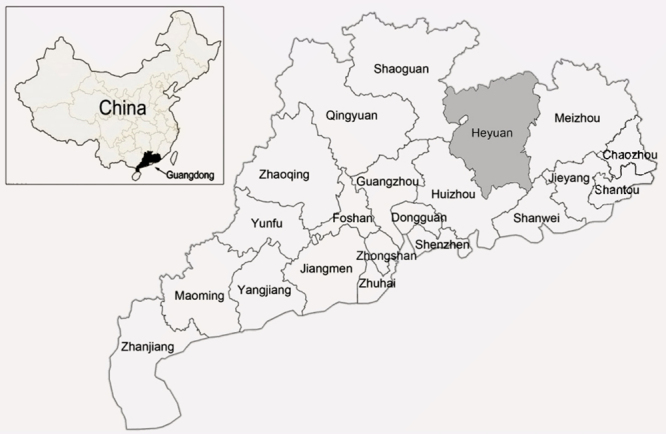
Geographic location of Heyuan city in Guangdong province.

According to investigation, the Hakkas with unique genetic characteristics are considered Han Chinese people that primarily live in southern China. Many studies have shown significant genetic heterogeneity among Hakka people in different regions (Zhao *et al*., 2018; Ma *et al*., 2021). As one of the primary residences of the Hakkas, Heyuan City is situated in the northern mountainous area of Guangdong Province, China (Figure 1). Knowingly, the occurrence of thalassemia in Guangdong Province is so high. However, the prevalence and molecular characterization of α-thal and β-thal mutations among Hakka people in Heyuan so far have not been reported. 

In this study, we retrospectively performed a survey on thalassemia genotypes among the Hakka population in Heyuan. This study aims to reveal the prevalence and molecular characterization of α- and β-thal mutations among Hakka people, provide the scientific basis for the prevention and control of thalassemia, and construct a detailed frequency map of the spectrum of thalassemia mutation in Heyuan. This study will provide comprehensive data on the prevalence of thalassemia in Heyuan, providing a valuable reference for the government to formulate thalassemia prevention and control policies.

## Material and Methods

### Study population

From October 2010 to August 2020, a total of 25,437 participants who came to the Heyuan Women and Children Hospital carried out α-thal and/or β-thal genetic testing. We eliminated 118 patients who were repeatedly tested in the inpatient or outpatient department. The age of the patients varied from a few days to 91 years old (median age: 24 years), and all the participants or their families obtained informed consent. This study was endorsed by the Medical Ethics Committee of the Heyuan Women and Children Hospital.

### Hematological analysis

The blood samples were obtained from the peripheral venous system of all subjects, which were collected in an anticoagulation tube containing ACD or EDTA-k_2_. Routine hematological examinations were performed using a hemocytometer (Sysmex XS-1800i, Japan). A capillary electrophoresis system (Sebia, France) was applied to analyze the levels of hemoglobin components. The subjects who met the following criteria were considered as suspected carriers of thalassemia and checked for further genetic analysis: (1) mean corpuscular volume (MCV) <82 fL and/or mean corpuscular hemoglobin (MCH) <27 pg; (2) HbA_2_ <2.5% or HbA_2_ >3.5% or HbF >2.0%; (3) abnormal haemoglobin zone; (4) parents or siblings carried the thalassemia gene mutation (Huang *et al*., 2019; Yang *et al*., 2019; Cai *et al*., 2021).

### DNA extraction and molecular analysis

The genomic DNA of suspected thalassemia subjects was obtained by the extraction kit (Zee san, China) and stored at -20 °C for subsequent tests. The concentration and purity of DNA were assessed by the NanoDrop 2000c UV-Vis spectrophotometer (Thermo Fisher, USA). The protocols are strictly following the manufacturer’s instructions. The deletion of α-thal mutation (--^SEA^/, -α^4.2^/, and -α^3.7^/) was tested by Gap-PCR. The PCR-RDB technique was applied to test the non-deletion α-thal mutations (α^CS^α/, α^QS^α/ and α^WS^α/), and 17 types of β-thal mutations, including IVS-Ⅱ-654(C>T), CD71-72(+A),CD17(A>T), CD43(G>T), -28(A>G), CD41-42(-TCTT), -29(A>G),βE (CD26,G>A), CD14-15(+G), -30(T>C), CAP(-AAAC), -32(C>A), CD27-28(+C), CD31(-C), IVS-Ⅰ-5(G>C), Int(ATG>AGG) and IVS-Ⅰ-1(G>A, G>T). The genetic analysis was done by commercial kits (Yaneng, Shenzhen, China), according to the manufacturer’s instructions (Munkongdee *et al*., 2020; Wang *et al*., 2021; Zhuang *et al*., 2021). Hkαα/-- ^SEA^ and Hkαα/αα was detected by nested PCR which was confirmed by Shenzhen Yaneng Biotechnology Co. Ltd.

### Statistical analyses

We used SPSS19.0 (IBM, USA) and Excel (Microsoft, USA) software to analyze the data. We used n% to express the constituent ratio. *P*<0.05 was used for a statistical cut-off.

## Results

### Genetic analyses of α-thal and β-thal

In the Heyuan region, the number of 25,437 suspected thalassemia subjects has further been indicated for genetic analysis of the thalassemia according to the effects of hematological analysis. Genetic studies revealed that 44.09% (11216/25437) subjects carried thalassemia variations, including 30.85% (7847/25437) with α-thal changes alone, 11.50% (2924/25437) with β-thal changes alone, and 1.75% (445/25437) subjects with both α-thal and β-thal mutations. In addition, 14221 cases (55.91%, 14221/25437) with thalassemia mutation were not found in the detection range.

### Distribution of α-thal and β-thal genotypes

As shown in Table 1, 23 genotypes were found in the 7847 α-thal carriers, among which the five common genotypes were --^SEA^/αα, -α^3.7^/αα, -α^4.2^/αα, α^CS^α/αα, --^SEA^/-α^3.7^, respectively, accounting for 68.34% (5363/7847), 16.44% (1290/7847), 6.38% (501/7847), 2.15% (169/7847), 2.14% (168/7847). There were 269 patients with Hb H among the 7847 α-thal carriers, including 217 patients of deletion Hb H and 52 patients of non-deletion Hb H. Interestingly, we detected six cases of the rare α-thal mutation, including two cases of HKαα/αα and four cases of HKαα/--^SEA^.

**Table 1:** Distribution of α-thal genotypes in Heyuan.

Types	Phenotypes	Genotypes	Total	Cases	Frequency (%)
‘Silent’α-thal	α^+^/α	-α^3.7^/αα	2147	1290	16.44%
	α^+^/α	-α^4.2^/αα		501	6.38%
	α^+^/α	α^CS^α/αα		169	2.15%
	α^+^/α	α^WS^α/αα		132	1.68%
	α^+^/α	α^QS^α/αα		55	0.70%
α-thal trait	α^0^/α	--^SEA^/αα	5425	5363	68.34%
	α^+^/α^+^	-α^3.7^/-α^3.7^		26	0.33%
	α^+^/α^+^	-α^4.2^/-α^3.7^		16	0.20%
	α^+^/α^+^	-α^3.7^/α^WS^α		6	0.08%
	α^+^/α^+^	-α^4.2^/-α^4.2^		5	0.06%
	α^+^/α^+^	-α^3.7^/α^CS^α		2	0.03%
	α^+^/α^+^	--α^4.2^/α^CS^α		2	0.03%
	α^+^/α^+^	α^WS^α/α^WS^α		2	0.03%
	α^+^/α^+^	-α^4.2^/α^QS^α		1	0.01%
	α^+^/α^+^	--α^4.2^/α^WS^α		1	0.01%
	α^+^/α^+^	α^QS^α/α^WS^α		1	0.01%
Hb H	α^0^/α^+^	--^SEA^/-α^3.7^	269	168	2.14%
	α^0^/α^+^	--^SEA^/-α^4.2^		49	0.62%
	α^0^/α^+^	--^SEA^ /α^CS^α		26	0.33%
	α^0^/α^+^	--^SEA^/α^WS^α		23	0.29%
	α^0^/α^+^	--^SEA^/α^QS^α		3	0.04%
the rare α-thal mutation	α^0^ /α^+^	HKαα/ --^SEA^	6	4	0.05%
	α^+^/α	HKαα/αα		2	0.03%
	**Total**		**7847**		**100%**

Among the 17 mutation sites of β-globin genes, we detected 13 kinds of mutation loci except for -30 (T>C), IVS-Ⅰ-5 (G>C), -32 (C>A), CD31 (-C). We found 29 kinds of genotypes, including 13 heterozygous (98.22%), 12 compound heterozygotes (1.33%), and four homozygotes (0.44%). β^CD41-42^/β^N^ is the top common genotype, accounting for 36.22% of the 2942 β-thal carriers, followed by β^IVS-Ⅱ-654^/β^N^(30.88%), β^-28^/β^N^(13.47%), β^CD17^/β^N^(8.93%), β^E^/β^N^(2.63%). Other genotypes were observed in less than 2% of β-thal carriers (Table 2).

**Table 2 -:** Distribution of β-thal genotypes in Heyuan.

Types	Phenotypes	Genotypes	Total	Cases	Frequency (%)
β-TT	β^0^/β^N^	β^CD41-42^/β^N^	2872	1059	36.22%
	β^+^/β^N^	β^IVS-Ⅱ-654^/β^N^		903	30.88%
	β^+^/β^N^	β^-28^/β^N^		394	13.47%
	β^0^/β^N^	β^CD17^/β^N^		261	8.93%
	β^+^/β^N^	β^E^/β^N^		69	2.36%
	β^0^/β^N^	β^CD14-15^/β^N^		52	1.78%
	β^0^/β^N^	β^CD27-28^/β^N^		52	1.78%
	β^0^/β^N^	β^CD71-72^/β^N^		29	0.99%
	β^+^/β^N^	β^-29^/β^N^		20	0.68%
	β^+^/β^N^	β^CAP^/β^N^		18	0.62%
	β^0^/β^N^	β^CD43^/β^N^		8	0.27%
	β^0^/β^N^	β^IVS-Ⅰ-1^/β^N^		5	0.17%
	β^0^/β^N^	β^Int^/β^N^		2	0.07%
β-TI	β^+^/β^+^	β^IVS-Ⅱ-654^/β^-28^	7	4	0.14%
	β^+^/β^+^	β^-28^ /β^-28^		2	0.07%
	β^+^/β^+^	β^-28^/β^CD26^		1	0.03%
β-TM	β^0^/β^+^	β^CD41-42^/β^IVS-Ⅱ-654^	45	13	0.44%
	β^0^/β^0^	β^CD41-42^/β^CD17^		5	0.17%
	β^0^/β^0^	β^IVS-Ⅱ-654^/β^IVS-Ⅱ-654^		5	0.17%
	β^0^/β^+^	β^CD41-42^/β^CAP^		4	0.14%
	β^0^/β^0^	β^CD41-42^/β^CD41-42^		3	0.10%
	β^0^/β^0^	β^CD17/^β^CD17^		3	0.10%
	β^0^/β^+^	β^CD41-42^/β^E^		2	0.07%
	β^0^/β^+^	β^CD41-42^/β^-28^		2	0.07%
	β^0^/β^+^	β^CD17^/β^-28^		2	0.07%
	β^0^/β^+^	β^CD27-28^/β^-28^		2	0.07%
	β^0^/β^+^	β^CD43^/β^IVS-Ⅱ-654^		2	0.07%
	β^0^/β^+^	β^CD14-15^/β^-28^		1	0.03%
	β^0^/β^+^	β^CD17^/β^IVS-Ⅱ-654^		1	0.03%
	**Total**			**2924**	**100%**

### Distribution of composite α-thal and β-thal mutation

In total, we determined 55 genotypes in the 445 subjects with composite α-thal and β-thal variations (Table 3). The top five genotypes of αβ-thalassemia were --^SEA^/αα combined with β^CD41-42^/β^N^(21.12%), --^SEA^/αα combined with β^IVS-Ⅱ-654^/β^N^(17.98%), --^SEA^/αα together with β^-28^/β^N^(9.66%), -α^3.7^/αα together with β^CD41-42^/β^N^(7.64%), -α^3.7^/αα together with β^IVS-Ⅱ-654^/β^N^(5.39%). The other 48 genotypes of α-thal combined with the β-thal mutation were infrequent, and the frequency does not exceed 5.0%. As shown in Table 3, the β-globin gene mutation (β^CD41-42^/β^N^, β^IVS-Ⅱ-654^/β^N^, β^-28^/β^N^, β^CD17^/β^N^) combined with the α-globin gene (--^SEA^/αα, -α^3.7^/αα, -α^4.2^/αα) made up of 78.20% of genotypes of αβ-thalassemia.

**Table 3 -:** Distribution of composite α-thal and β-thal in Heyuan.

β‐thalassemia variations	α‐thalassemia variations										Total
	--^SEA^/αα	-α^3.7^/αα	-α^4.2^/αα	α^WS^α/αα	--^SEA^/-α^3.7^	α^CS^α/αα	--^SEA^/-α^4.2^	α^QS^α/αα	-α^3.7^/-α^3.7^	HKαα/--^SEA^	
β^CD41-42^/β^N^	94	34	13	7	4	3	1	1	1	1	**159**
β^IVS-Ⅱ-654^/β^N^	80	24	11	9	4	1	0	2	1	0	**132**
β^-28^/β^N^	43	17	2	2	2		0	0	0	0	**66**
β^CD17^/β^N^	17	11	2	2	1	1	0	0	0	0	**34**
β^CD14-15^/β^N^	5	4	2	0	0	0	0	0	0	0	**11**
β^E^/β^N^	11	3	0	0	1	0	3	0	0	0	**18**
β^CD27-28^/β^N^	2	2	1	1	0	0	0	0	0	0	**6**
β^CAP^/β^N^	4	0	0	0	0	1	0	0	0	0	**5**
β^CD71-72^/β^N^	1	1	1	0	0	0	0	0	0	0	**3**
β^-29^/β^N^	2	0	0	0	0	0	0	0	0	0	**2**
β^Int^/β^N^	1	0	0	0	0	0	0	0	0	0	**1**
β^-28^/β^IVS-Ⅱ-654^	1	0	0	0	0	0	0	0	0	0	**1**
β^CD41-42^/β^IVS-Ⅱ-654^	1	0	0	0	0	0	0	0	0	0	**1**
β^IVS-Ⅰ-1^/β^N^	1	0	0	0	0	0	0	0	0	0	**1**
β^IVS-Ⅱ-654^/β^CAP^	1	0	0	0	0	0	0	0	0	0	**1**
β^CD41-42^/β^-28^	1	0	0	0	0	0	0	0	0	0	**1**
β^CD43^/β^N^	1	0	0	0	0	0	0	0	0	0	**1**
β^-28^/β^CD14-15^	0	1	0	0	0	0	0	0	0	0	**1**
β^CD17^/β^IVS-Ⅱ-654^	0	1	0	0	0	0	0	0	0	0	**1**
**Total**	**266**	**98**	**32**	**21**	**12**	**6**	**4**	**3**	**2**	**1**	**445**

### Allele frequencies of α-thal and β-thal mutations

The allele frequencies of α-globin gene variation are shown in Table 4. The results revealed that 8646 chromosomes with α-thal gene variation were detected, including six α-thal gene mutations. For these mutations, the top rates of α-thal gene mutation were --^SEA^, accounting for 68.46% of all α-thal variation chromosomes, followed by -α^3.7^(19.06%), -α^4.2^(7.12%), α^CS^α(2.37%), α^WS^α(2.17%), α^QS^α(0.73%) and HKαα(0.08%). There were 3426 chromosomes with β-thal gene variation, including 13 β-thal gene variation. For these mutations, the top rates of β-thal gene mutation were CD41-42(-TCTT)(36.54%), followed by IVS-II-654(C>T)(31.20%), -28(A>G) (13.98%), CD17 (A>T) (9.05%), βE (CD26,G>A) (2.60%), CD14-15(+G) (1.90%), CD27-28(+C) (1.75%), CD71-72(+A) (0.93%), CAP(-AAAC) (0.82%), -29(A>G) (0.64%), CD43(G>T) (0.32%), IVS-Ⅰ-1(G>T, G>A) (0.18%), and Int(ATG>AGG) (0.09%) (Table 4).

**Table 4 -:** Allele frequencies of α-thal/β-thal mutations in Heyuan.

Type	Mutation	Phenotype	Allele (n)	Allele Frequency (%)
α-thal	--^SEA^	α^0^	5919	68.46
	-α^3.7^	α^+^	1648	19.06
	-α^4.2^	α^+^	616	7.12
	α^CS^α	α^+^	205	2.37
	α^WS^α	α^+^	188	2.17
	α^QS^α	α^+^	63	0.73
	HKαα	α^+^	7	0.08
	**Total**		**8646**	**100**
β-thal	CD41-42(-TCTT)	β^0^	1252	36.54
	IVS-Ⅱ-654(C>T)	β^+^	1069	31.20
	-28(A>G)	β^+^	479	13.98
	CD17(A>T)	β^0^	310	9.05
	βE (CD26, G>A)	β^+^	89	2.60
	CD14-15(+G)	β^0^	65	1.90
	CD27-28(+C)	β^0^	60	1.75
	CD71-72(+A)	β^0^	32	0.93
	β-29(A>G)	β^+^	22	0.64
	CAP(-AAAC)	β^+^	28	0.82
	CD43(G>T)	β^0^	11	0.32
	IVS-Ⅰ-1(G>T, G>A)	β^0^	6	0.18
	Int(ATG>AGG)	β^0^	3	0.09
	**Total**		**3426**	**100**

## Discussion

This is the first large-scale research to assess the prevalence and molecular properties of thalassemia mutation among Hakka people in Heyuan. We discovered that the total incidence of thalassemia mutation carriers was 44.09%, including 30.85% subjects of α-thal variation alone, 11.50% subjects of β-thal variation alone, and 1.75% subjects of both of them. It is much higher than that of the neighboring regions Shaoguan (Ma *et al*., 2021) and Meizhou (Zhao *et al*., 2018). There were signiﬁcant statistical differences between the three regions which are located in Guangdong province (*P*<0.01). Some possible reasons may explain this difference. It may be because, firstly, we selected the subjects who were primarily suspected of thalassemia, while the subjects were used for routine health examination in other studies. Besides, ethnic differences are also noted. In Shaoguan and Meizhou, ethnic minorities such as Yao people and She people are included (Zhao *et al*., 2018; Ma *et al*., 2021). In our study, only the Hakka people of Han nationality are studied.

Our studies indicated that the five prominent genotypes in the 23 identified genotypes were --^SEA^/αα, -α^3.7^/αα, -α^4.2^/αα, α^CS^α/αα, and --^SEA^/-α^3.7^, which are composed of 95.45% of all the α-thal mutation carriers. It is similar to the situation in the Hakka people of Meizhou, which is adjacent to Heyuan. This is also consistent with the --^SEA^ mutation which is commonly found in thalassemia variants in the Southeast. Whereas, α^QS^α/αα had a low detection rate in the non-deletion α-thal mutations, which was consistent with the previous study in Guangxi province (Lai *et al*., 2017; He *et al*., 2017; He *et al*., 2018). Compatible with the overall detection of α-thal genotype, --^SEA^, -α^3.7^, -α^4.2^, α^CS^α are the top allele frequencies of α-thal mutations in Heyuan. 

A large amount of data shows that there are great differences of β-thal mutations in different regions and different races. IVS1-5 (G > C) and CD41/42 (-TTCT) are the most commonest in South Asia and Southeast Asia, respectively. In Heyuan, the detection of 29 genotypes and 13 kinds of mutation loci displayed the molecular characterization of β-thal among the Hakkas people. The typical five genotypes are β^CD41-42^/β^N^(36.22%), β^IVS-Ⅱ-654^/β^N^(30.88%), β^-28^/β^N^(13.47%), β^CD17^/β^N^(8.93%), and β^E^/β^N^(2.36%), which were compatible with gene frequencies of β-thal mutations in Heyuan. β^CD41-42^/β^N^ is the top common genotype, which was analogous to the research published in the Guangxi provinces (Lai *et al*., 2017; He *et al*., 2018). Nevertheless, β^IVS-Ⅱ-654^/β^N^ is the foremost common β-thal variation in other regions, such as Meizhou (Zhao *et al*., 2018), and the Chaoshan region (Zheng X *et al*., 2016) of Guangdong provinces, Hubei provinces (Zhu *et al*., 2020). It is worth noting that the Hakka people are the main subjects studied in Heyuan and Meizhou, but the results of β-thal mutation are different. This needs further research to verify.

Additionally**,** up to 445 subjects with composite α-thal and β-thal variations were detected in our study, including 16 with Hb H disease compound β-thal. But in another Hakka region (Zhao *et al*., 2018), among the 148 cases of composite α-thal and β-thal variations, only two examples were Hb H disease compound β-thal. We also detected seven cases of HKαα mutation, including six subjects of heterozygous mutation and one subjects of HKαα compound β-thal. The above results suggested that all patients of β-thal carriers should be carefully screened for α-thalassemia to obtain the correct genotype, and to carry out proper risk assessment and consultation, especially during prenatal diagnosis. 

Building on our results, the pressure of prevention and control for thalassemia in Heyuan should not be underestimated. Heyuan is a relative poverty-stricken mountainous region in Guangdong province. According to data from the People’s Government of Heyuan in 2020 (http://www.heyuan.gov.cn/), the per capita disposable income of urban residents and the per capita disposable income of rural residents are ¥2,8018.2 and ¥1,7313.4, respectively. Patients with β-TM need regular red blood cell transfusion and iron chelation therapy to maintain their lives, but the annual cost is about ¥10,000. Another issue is that due to the high frequency of --^SEA^ thalassemia in Heyuan, Hb Bart’s hydrops fetalis is harmful to the mother. 

According to our data, there are certain peculiarities about molecular characteristics of thalassemia gene variation among the Hakka population in Heyuan. It may have something to do with the Hakka, who fuse the genetic aspects of the Han people in both northern and southern China (Zheng L *et al*., 2013). Although we have obtained some exciting discoveries in our research, there are still some shortcomings. Firstly, the major limitation of this study are the diagnostic capabilities for rare thalassemia types are insufficient apart from HKαα. With the development of technology, it will be possible to fully apply molecular diagnostic technology to discover unique kinds of thalassemia. On the other hand, we just retrospectively assessed the molecular characteristics of genetic testing for thalassemia, without prenatal diagnosis and its follow-up. In the future, we should strengthen the analysis of additional clinical data to provide a more dependable reference for clinical consultation.

In conclusion, this study has indicated that --^SEA^/αα and β^CD41-42^/β^N^ are the most frequent genotypes of α- and β-thal mutations among Hakka people in Heyuan, respectively. Besides, this research has displayed that the types of thalassemia mutations among the Hakka population in Heyuan have a high degree of heterogeneity and broad-spectrum. These greatly enriched the spectrum of thalassemia mutation in Chinese people. These results have important practical significance for carrying out effective clinical consultation and preventing the occurrence of TM in the Heyuan region.
